# Musculoskeletal Progenitor/Stromal Cell-Derived Mitochondria Modulate Cell Differentiation and Therapeutical Function

**DOI:** 10.3389/fimmu.2021.606781

**Published:** 2021-03-08

**Authors:** Christian Jorgensen, Maroun Khoury

**Affiliations:** ^1^ Inserm, U1183, Montpellier, France; ^2^ Université MONTPELLIER 1, UFR de Médecine, Montpellier, France; ^3^ Service d’immuno-Rhumatologie, Hôpital Lapeyronie, Montpellier, France; ^4^ Laboratory of Nano-Regenerative Medicine, Centro de Investigación e Innovación Biomédica (CIIB), Faculty of Medicine, Universidad de los Andes, Santiago, Chile; ^5^ Cells for Cells, Santiago, Chile; ^6^ Consorcio Regenero, Chilean Consortium for Regenerative Medicine, Santiago, Chile

**Keywords:** musculoskeletal progenitor/stromal cells, immunosuppression, mitochondria, stem cell, immunometabolism

## Abstract

Musculoskeletal stromal cells’ (MSCs’) metabolism impacts cell differentiation as well as immune function. During osteogenic and adipogenic differentiation, BM-MSCs show a preference for glycolysis during proliferation but shift to an oxidative phosphorylation (OxPhos)-dependent metabolism. The MSC immunoregulatory fate is achieved with cell polarization, and the result is sustained production of immunoregulatory molecules (including PGE2, HGF, IL1RA, IL6, IL8, IDO activity) in response to inflammatory stimuli. MSCs adapt their energetic metabolism when acquiring immunomodulatory property and shift to aerobic glycolysis. This can be achieved *via* hypoxia, pretreatment with small molecule-metabolic mediators such as oligomycin, or AKT/mTOR pathway modulation. The immunoregulatory effect of MSC on macrophages polarization and Th17 switch is related to the glycolytic status of the MSC. Indeed, MSCs pretreated with oligomycin decreased the M1/M2 ratio, inhibited T-CD4 proliferation, and prevented Th17 switch. Mitochondrial activity also impacts MSC metabolism. In the bone marrow, MSCs are present in a quiescent, low proliferation, but they keep their multi-progenitor function. In this stage, they appear to be glycolytic with active mitochondria (MT) status. During MSC expansion, we observed a metabolic shift toward OXPhos, coupled with an increased MT activity. An increased production of ROS and dysfunctional mitochondria is associated with the metabolic shift to glycolysis. In contrast, when MSC underwent chondro or osteoblast differentiation, they showed a decreased glycolysis and inhibition of the pentose phosphate pathway (PPP). In parallel the mitochondrial enzymatic activities increased associated with oxidative phosphorylation enhancement. MSCs respond to damaged or inflamed tissue through the transfer of MT to injured and immune cells, conveying a type of signaling that contributes to the restoration of cell homeostasis and immune function. The delivery of MT into injured cells increased ATP levels which in turn maintained cellular bioenergetics and recovered cell functions. MSC-derived MT may be transferred *via* tunneling nanotubes to undifferentiated cardiomyocytes and leading to their maturation. In this review, we will decipher the pathways and the mechanisms responsible for mitochondria transfer and activity. The eventual reversal of the metabolic and pro-inflammatory profile induced by the MT transfer will open new avenues for the control of inflammatory diseases.

## Introduction

Musculoskeletal pogenitor/stromal cells (MSCs) (also referred to as mesenchymal stem cells) have been proposed as a cell therapy for mesoderm-derived tissue regeneration and immune modulation. MSCs are the progenitors of mesoderm lineages including bone, cartilage, muscle, fat, tendon, and synovium. MSCs can be isolated from the bone marrow (BM) (1), adipose tissue (Ad) (2), umbilical cord blood (UCB), placenta, Wharton’s jelly (3), dental pulp (4), or from induced pluripotent stem (IPS) cell-derived MSCs (5). Due to easy access and high productivity, autologous and allogeneic MSCs are currently most often obtained from bone marrow or adipose tissue, while allogeneic MSCs are also obtained from UCB or Wharton’s jelly (3). Significant functional differences between MSC sources have been reported. The best strategy for MSC-based therapy has therefore to be determined according to their distinct characteristics associated with their tissue origin for a particular therapeutic application.

Variation in MSC sources, passage number during *ex vivo* culture, and age-related fatigue of MSC function and change of cell metabolism during expansion may result in variability in MSC function (6). This may induce heterogeneity in clinical trial results. Understanding of regulation of MSC metabolism is critical as that the manipulation of cell metabolism allows enhanced therapeutic uses of these cells (*e.g.*, cell retention, cell survival, immunoregulation, differentiation) in cell-based medicine and tissue engineering. Here, we review the present knowledge on the metabolic pathways involved in the function of MSC and the perspectives for their optimal therapeutic applications.

## Energetic Metabolism Is Critical In MSC Differentiation and Function

**Figure 1:**
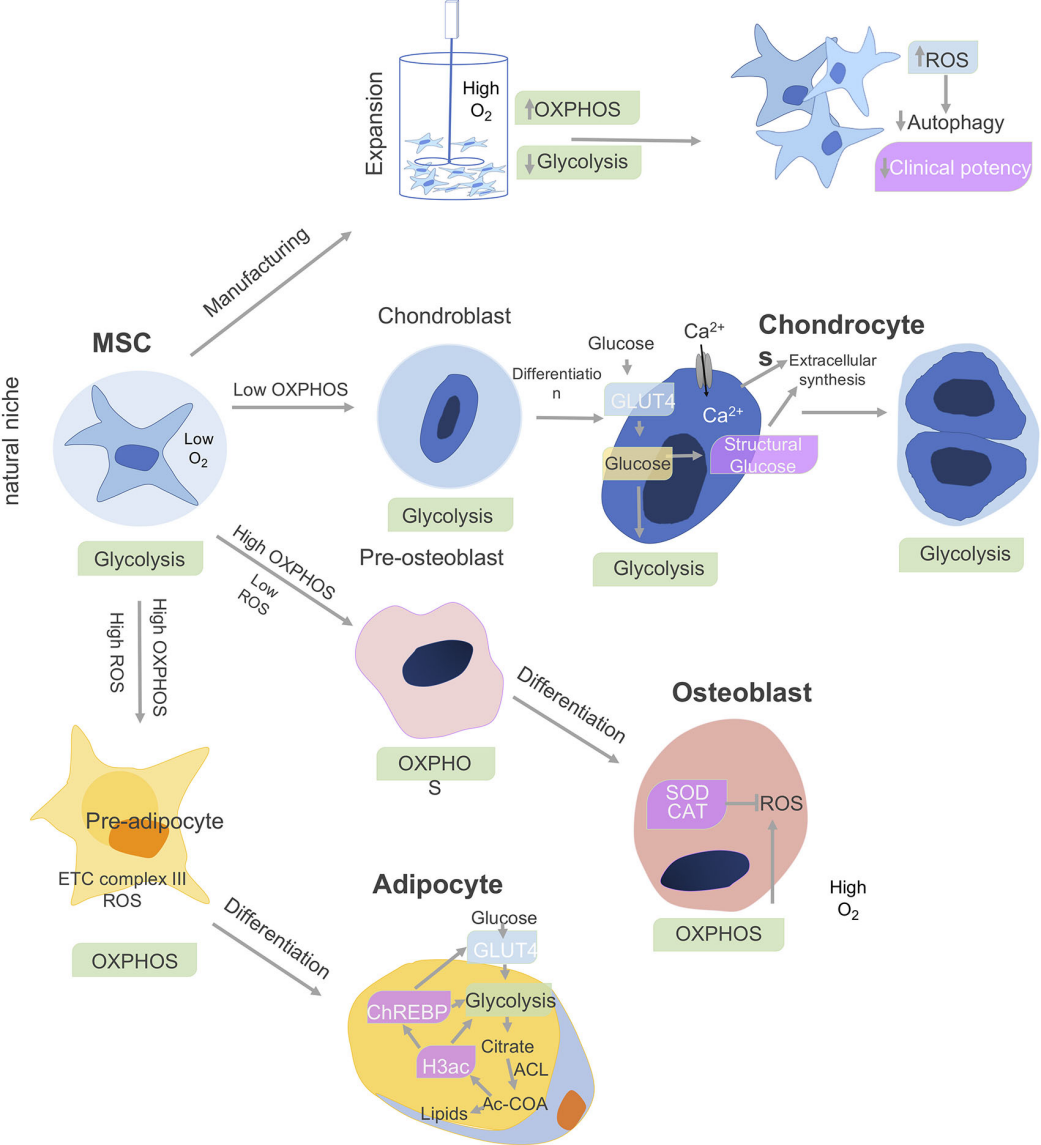
Cell metabolism and MSC differentiation. The metabolism of MSCs changes depending on the niche and differentiation stages. The bone marrow environment is a hypoxic niche where MSCs remain quiescent and use glycolysis. When isolated and expanded *ex vivo* in a nutrient-rich artificial culture environment, proliferation is promoted, which derives a significant proportion of ATP from oxidative phosphorylation (OXPHOS) in addition to glycolysis. The accumulation of cytotoxic metabolic byproducts, including reactive oxygen species (ROS), reduces basal autophagy and mitophagy rates while increasing the fraction of senescent cells leading to reduced clinical potency. Under chondro-inductive differentiation conditions, glycolysis is further upregulated during chondrogenesis in chondroblasts and which gives rise to chondrocytes and cartilage. In osteoblasts, which give rise to bone, both O_2_ consumption and oxidative phosphorylation (OXPHOS) are upregulated, while superoxide dismutase (SOD) and catalase (CAT) negatively regulate ROS secretion. Finally, adipocyte differentiation is associated with upregulation of OxPhos metabolism, release reactive oxygen species (ROS), and electron transport chain (ETC) complex III. Adipocytes upregulate glycolysis and ATP citrate lyase (ACL), which leads to increased cytosolic acetyl-CoA (Ac-CoA) synthesis. In parallel, increase in histone H3 acetylation (H3ac) is associated with lipid synthesis. H3ac activates the carbohydrate-responsive element-binding protein (ChREBP) transcription factor to promote glycolysis through GLUT4-mediated glucose uptake (9).

MSC metabolism impacts cell differentiation as well as immune function. BM-MSCs show a preference for glycolysis during proliferation. During proliferation, the human MSCs (hMSCs) primarily generate ATP through glycolysis (7) but shift to oxidative phosphorylation (OxPhos)-dependent metabolism during osteogenic and adipogenic differentiation (OD, AD) (8), as depicted in **Figure 1**. In contrast, adipose derived ASC increased both glycolysis and mitochondrial metabolism associated with OxPhos and fatty acid b-oxidation (10). When Ad-hMSCs underwent adipogenesis, they showed a decreased capacity for the pentose phosphate pathway (PPP) and glycolysis, while mitochondrial enzyme activities increased in parallel to oxidative phosphorylation and b-oxidation (**Figure 1**).

To induce immune stimulation and polarization in hMSCs, activation through proinflammatory cytokine including TNFa and/or IFN-g is necessary. In response to this inflammatory stimuli, MSCs demonstrate immunoregulatory function associated with cell polarization and enhanced production of immunoregulatory factors (including PGE2, HGF, IL1RA, IL6, IL8, IDO activity) (11, 12). Interestingly, this MSC polarization requires a metabolic shift toward aerobic glycolysis.

Moreover, hypoxia enhanced MSC immunomodulatory function through a change in cell metabolism. Similar change in cell metabolism is obtained with pretreatment with small molecule-metabolic mediators such as oligomycin or AKT/mTOR pathway modulation. The immunoregulatory effect of MSC on macrophage polarization and Th17switch is related to the glycolytic status of the MSC. MSCs pretreated with oligomycin decreased the M1/M2 ratio, inhibited T-CD4 proliferation, and prevented Th17 switch (13).

After cytokine stimulation, hMSC metabolism switches their metabolic pathways toward glycolysis (reducing TCA cycle metabolism), and this seems to be required for a sustained immunosuppressive effect (14). PGE2 and IDO activity increased in response to augmented aerobic glycolysis. Consequently, the hMSC activation was associated with increased expression of glucose transporter 1 and hexokinase isoform 2 (key enzymes in glycolysis), and reduced electron transport and OxPhos. Furthermore, activated MSCs exhibited a glycolytic phenotype and inhibited activity of the mitochondrial electron transport chain (complexes I or III), blocking OxPhos and reducing mitochondria-related reactive oxygen species (mROS) (14). The Akt/mTOR signaling pathway activation was shown to be key in the induction of metabolic switch (15). Through induction of mTOR activity, IFN increases the glycolytic capacity of the MSC. This energy metabolic modification enhances the MSC ability to inhibit T cell proliferation. As a result, the IFN-g induced expression of growth factors, including hepatocyte growth factor (HGF), that is involved in immune and antifibrotic functions of hMSCs (16).

## MSC Expansion and Alteration in The Metabolism

To achieve sufficient numbers of MSCs for clinical transfer, cell expansion is a necessary step. This cell expansion is performed either in flask with a tight control of oxygen and nutriment and CO_2_ level or in bioreactors. In the bone marrow, hMSCs are present in a quiescent state with low cell proliferation maintained overtime. In this undifferentiated fate, MSCs are in glycolytic metabolism with small mitochondria maintained by active autophagy and mitophagy (17). Inducing hMSC proliferation in a bioreactor leads to an enhanced significant ATP production derived from oxidative phosphorylation (OXPHOS) to support the proliferation (18). This results in accumulation of reactive oxygen species (ROS), reduction of the basal autophagy rate and dysfunctional mitochondria, and increase of senescent cells (19, 20). As the MSC function is associated with glycolytic state, bioreactor expansion leads to emergence of OXPHOS metabolic profile MSC with reduction if differentiation function (21) is associated with the accumulation of reactive oxygen species (ROS) (9). This leads to cell senescence and reduction in autophagy and mitophagy rates (21).

## Regulation of MSC Metabolism to Improve Cell Function

Hypoxia enhances hMSC immunoregulatory properties. In a GVHD preclinical study, hypoxic pretreatment of hMSC enhanced the secretion of IL-10 and Fas ligand and improved animal survival and weight loss (22). Overexpression of HIF1-a mimics hypoxia and is shown to enhance hMSC immunomodulatory properties (13). Preconditioning of the cells with N-acetylcysteine (NAC), which stabilizes HIF-1, was shown to improve MSC anti-inflammation cell retention (23), and osteogenesis (24).

Pre-activation of MSCs with IFN has been widely reported to enhance MSC immunomodulatory properties as well as an inducer of the metabolic switch in preclinical models. For example, the infusion of IFN-g pretreated MSCs in an immunodeficient mouse model significantly reduced the symptoms of GVHD and improved survival (25).

Recently, we have shown that PPAR*β*/*δ* is critical for the immunoregulatory functions of MSC. The upregulation of this transcription factor enhanced the expression of genes associated with fatty acid transport and *β*-oxidation (26). PPAR*β*/*δ* increases MSC glycolytic activity required for their immune impact on Th1 and Th17 cells (26). Moreover, PPAR*β*/*δ* inhibited mitochondrial ATP production and promoted their metabolic switch towards aerobic glycolysis leading to enhancement of their immunosuppressive functions. Preconditioning MSC with PPAR*β*/*δ* inhibitors improved MSC function through metabolic reprogramming and enhanced MSC immunoregulatory properties in arthritis models (26).

On the other hand, Notch signaling is a conserved pathway that regulates cell-fate determination during development and maintains adult tissue homeostasis. The activation of Notch by either Jagged1 or the Notch2 intracellular domain suppresses glucose metabolism in mesenchymal progenitors and inhibits their osteoblastic differentiation potential. AMP-activated protein kinase (AMPK) plays a critical role as regulator of cellular metabolic homeostasis. Notch downregulated AMPK activity and inhibited glycolysis in hMSC (27). Notch reduces the expression of mitochondrial complex I genes, resulting in a decrease in mitochondrial respiration, superoxide production. Thus Notch pathway prevented osteoblastogenesis from bone marrow mesenchymal progenitors, demonstrating that AMPK is critical for MSC differentiation potential (27).

The mechanistic Target of Rapamycin (mTOR) coordinates eukaryotic cell growth and metabolism with environmental inputs including nutrients and growth factors.

mTORC1 facilitates growth by promoting a shift in glucose metabolism from oxidative phosphorylation to glycolysis, which likely facilitates the incorporation of nutrients into new biomass (15). Rapamycin is an mTOR inhibitor used to precondition MSCs to upregulate their immunosuppressive functions. Rapamycin inhibited cyclooxygenase (COX)-2 and PGE2 expression. Rapamycin preconditioning of MSCs strongly inhibited Th17 cell expansion through the upregulation of IL-10, IDO, and TGF-*β* (28). This confirmed that mTOR inhibition enhanced the therapeutic effect of MSCs in autoimmune diseases.

## Mitochondria Transfer, A New MSC Intercellular Modulatory Pathway

The requirement of cell-contact for MSCs to display immunoregulatory abilities is convergent with a set of recent observations that indicates that these cells can also achieve a range of effects by means of the transfer of their own mitochondria (MT) (29), to target renal, myocardial, or lung alveolar cells to restore target tissue homeostasis (30, 31). Thus, MSCs can also transfer this organelle to immunocompetent cells, conveying a type of signaling that contributes to the restoration of immune homeostasis (32). It is of considerable interest to elucidate this additional regulatory mechanism, potentially underlying immune function of MSCs.

The clinical significance of this phenomenon was first assessed in a model of lipopolysaccharide (LPS)-induced lung injury in which the intra-tracheal administration of MSCs to LPS treated mice was associated with the transfer of MT (MitoT) to the alveolar epithelium. MSCs triggered an increase in the concentration of ATP, metabolic activity and also an improvement in lung damage while reducing mortality in the diseased animals. The effects were dependent on the MSC expression of connexin 43 (CNX43) and the generation of nanotubes bridging alveolar cells (33). Subsequently, in an allergen mediated model of bronchial inflammation, MitoT from MSCs was shown to be controlled by Miro1, a MT Rho-GTPase (34). Again, MSC treatment reversed airway inflammation, hyper responsiveness and tissue damage. Exogenously administered MSCs were found to respond to tissular oxidative stress, rescuing epithelial cells by a process of MitoT that occurred *via* microtubules forming tunneling nanotubes (TNT) between cells. A set of elegant experiments showed these structures were the conduit responsible for the transportation of organelles by bridging MSCs with their target cells when submitted to stress (34). In another model of MSC co-cultured with vascular endothelial cells (VECs), the latter were able to induce proliferation of MSCs, a phenomenon partially suppressed with an inhibitor of TNT or by using VECs with defective MT (35). The relevance of MitoT from MSCs was also demonstrated in a model of ethidium bromide induced damage of MT in a cancer cell line. Treated cancer cells had reduced metabolic and proliferative capacity under standard conditions, an effect that was reversed by co-culture with MSCs. The rescue was linked to a restoration of the synthesis of nucleotides, found to be impaired by lack of functional MT in the cancer cell lines (36). More recently the transfer of MT from MSCs to macrophages has also been described. Jackson et al. observed that MT transferred from MSCs were able to increase the phagocytic capabilities of macrophages, increasing basal respiration and ATP turnover *in vitro* and *in vivo* in a murine model of acute respiratory distress syndrome (ARDS) (37). The mechanism by which MSCs transferred MT in this case was independent of CNX43 but related to the establishment of TNT between both cell types. Interestingly, the artificial transfer of MT from MSCs into macrophages conveys the same metabolic and phagocytic improvement, evidencing a role for MitoT in macrophage function (REF) (37). To date, it is well established that MSCs suppress T cell function (38). Given the relevance of the Th1/Th17 and Treg balance in the outcome of immune mediated diseases and the participation of MSCs in controlling this balance by means of cell-to-cell contact, MT transfer from MSCs to CD4+ T cell subpopulations plays a role in the regulation of T cell function, contributing to the dampening of inflammation that is associated with MSC therapy in graft *versus* host disease (GVHD) and other immune mediated conditions (39). Studies from our group showed immunosuppressive effects of MitoT from MSCs to lymphocytes, promoting Treg differentiation and inhibiting th17 (40). In an experimental xeno-GVHD model, we showed that MT isolated from MSCs and transferred to human PBMCs before their infusion alleviates inflammatory responses leading to significant improvement in the survival and reduction in tissue damage and organ T CD4+, CD8^+^, and IFN‐*γ* ^+^ expressing cell infiltration (41). These results represent strong evidence in favor of the hypothesis that the transfer of MSC-derived MT to immune active cells could play a role in the control of immune function mediated by MSCs.

## MSC Transfer Their MT Mainly Through Tunneling Nanotubes

**Figure 2:**
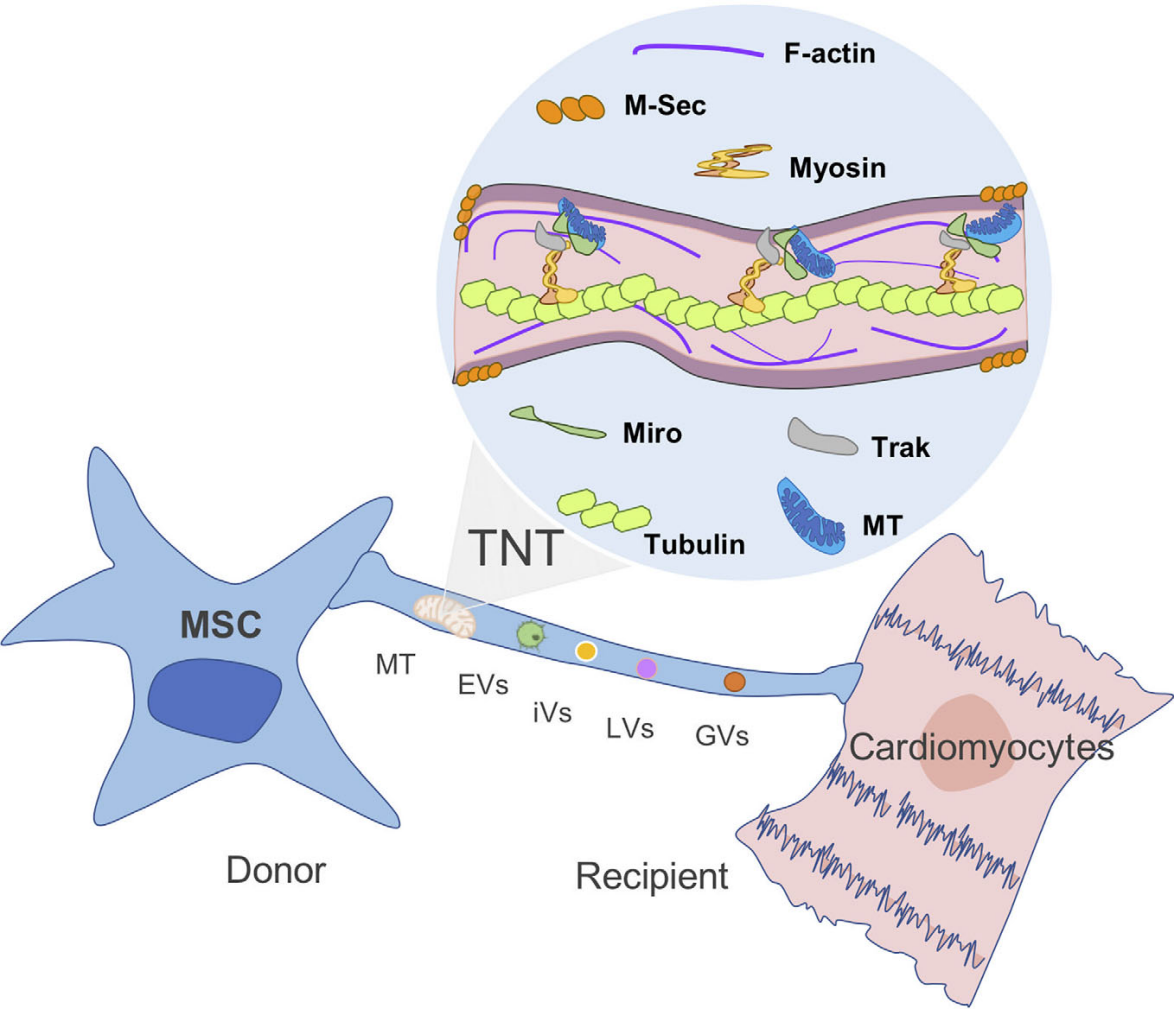
Mitochondria transfer from MSC to recipient cells through TNT. *Tunneling nanotubes (TNTs)* are small membranous thin cytoplasmic extensions bordered by a plasma membrane and connecting cells of 50–1,000 nm in diameter, containing both F-actin and microtubules. M-sec, a mammalian protein, induces formation of TNTs that only contain actin filaments, but without microtubules. Rho GTPases play an important role in mitochondrial motility through TNT. For instance, Miro1 and microtubules are involved in the regulation of organelle transfer, while Track and Myosin aid to move the mitochondria through the filament (44, 45, 46). MT, mitochondria; EVs, extracellular vesicles; iVs, intracellular vesicles; LVs, lysosomal vesicles; and GVs, Golgi vesicles (GVs).

Even though mitochondrial exchange has been well reported in the literature, the underlying mechanisms and signaling pathways associated with MT transfer remain to be elucidated. Upon injury, a distress signal is released by damaged cells that can be picked up by surrounding MSCs through their surface “sensors”, triggering an immediate mobilization and a migration shift toward the injury site. The MSC that migrated to the injured tissue will release growth factors as well as IDO activity in response to the microenvironment stimulus. The role of MSCs in tissue homeostasis is also associated with organelle exchange to damaged recipient cells (42). The formation of channels and membrane-like structures that allow the transfer of MT between MSCs and the recipient cells has been visualized and described as tunneling nanotubes (TNTs), vesicles, gap junctions, and fusion (37, 43). As described by Rustom et al. TNTs are long-distance tubular structures or projections that have a diameter between 50 and 150 nm and are able to connect different cells together (44). TNTs do not attach to the substrate of the cell nor are tethered to the extracellular matrix (**Figure 2**). Release and donation are not limited to MT as other organelles and cellular structures can also transit including extracellular and intracellular vesicles, Golgi, and lysosomal vesicles in a highly well-orchestrated process. The formation of TNTs and organelle motility inside these structures are well-regulated process by proteins known as TNFAIP2 and Miro1 along with accessory proteins, such as Miro2, TRAK1, KHC, and Myo19. The inhibition of Miro1 in MSC does not prevent the formation of TNT but retards mitochondrial movement, showing that mitochondrial donation is a well-directed process and not only a general exchange of cellular contents (34, 45, 47–49). This method appears to be essential for the transfer of mitochondria in some cell types including MSC, as chemical impairment of the structures reduces significantly mitochondrial exchange (50).

Another mechanism by which MSCs exert their therapeutic effects on recipient cells is *via* Extracellular Vesicles (EVs) and apoptotic bodies (51). While Phinney et al. studied the effects of MSCs on intracellular stress response, showing that MSCs prompt the movement of depolarized mitochondria into the outer limits of the plasma membrane as a response to a higher concentration of oxygen, in EVs larger than 100  nm (17). These EVs were loaded with mitochondria and secreted *via* arrestin domain-containing protein 1 and can fuse with macrophages, enhancing their oxygen consumption rate (17, 52, 53). The mechanisms of transfer that do not rely on long distance or secreted structures have also been reported in literature. For instance, Oh et al. has reported cell fusion on a model of myocardial infarction after stem cell therapy between cardiomyocytes and bone marrow transplanted cells (54). On the other hand, gap junctions are a processed by which MSCs attached to cells in regions of high connexin expression, for molecule and organelle exchange (46). Indeed, BMSCs formed Connexin43-mediated gap junctional channels with the alveolar epithelium, which allowed the transfer of mitochondria that led to cellular protection upon infection (46).

## Artificial Transfer to Selectively Assess The Functional Effect of MSC-Derived MT

Caicedo et al. used the term of MitoCeption to define the artificial way to transfer MT from a donor into a recipient cell. MSCs derived active mitochondria (MT) were transferred into a MDA-MB-231 cancer cell line. The “mitocepted” cells were able to increase glycolytic metabolism as well as their ATP production. Moreover, the recipient MDA cells recovered invasive and proliferative capacities (55). Another way to transfer MT has been described based on a so called “photothermal nanoblade”, independent of endocytosis and cell fusion. The nanoblade rescued the pyrimidine auxotroph phenotype and respiration of *ρ*0 cells that lack mtDNA (56). Of interest, metabolomic analysis of the mitocepted cells showed a change that occurs in the mtDNA haplotype in receptor somatic mammalian cells, suggesting this might be a procedure to treat MT genetic defects. Artificial transfer selectively allows the analysis of the sole impact of MT from MSCs to a specific target cell, without the interference of other parameters such as cell contact and paracrine factors.

## Future Perspective For Enhanced MSC-Dependent MT Transfer

Different strategies to augment MSC-MT biomass can be considered to reinforce their MT donation potential. This includes their *ex vivo* pretreatment with specific drugs that can activate AMPK (AMP-activated protein kinase) and the downstream signaling molecules including PGC-1alpha (peroxisome-proliferator-activated receptor gamma co-activator-1alpha) resulting in increasing MT biogenesis (57, 58).

Recently, MT were isolated from donor BMSCs and transferred into recipient BMSCs of the same batch and passage (59). The metabolically augmented MSCs through receiving autologous MT exhibited a significantly enhanced proliferation and migration; more importantly, following osteogenic induction, they showed an increased osteogenesis potential through the upregulation of the aerobic metabolism. The transplantation of the modified MSCs into a rat cranial critical-size bone defect model showed an improved bone formation *in situ*. Increased OXPHOS activity and ATP production were observed, which upon inhibition by oligomycin attenuated all the enhancement functions including the osteogenic differentiation (59).

Mitochondrial diseases are rare genetic disorders that occur when the mitochondria fail to produce enough energy for cell function (60). Mitochondrial diseases can affect almost any tissues, including the neurons (61), myocytes (62), kidney (63). Depending on which cells and tissue type are affected, symptoms may include loss of motor control, muscle weakness and pain, gastrointestinal disorders, intestinal malabsorption syndrome, growth retardation, heart disease, diabetes, visual or hearing impairment, susceptibility to infections, fertility and hormonal disorders, among many other pathologies. Since mitochondria come only from the maternal oocyte these inherited mitochondrial diseases are exclusively matrilineal. Moreover, not all mitochondria are mutated (mitochondrial heteroplasmy), and severity of symptoms depend on the percentage of mutant mitochondria in cells. In these rare disorders, transfer of mitochondria after expansion of donor cells, isolation of organelles and reinfusion through vector or physical cell introduction could be a therapeutical options (64). However, before clinical mitochondrial transfer is applied, many questions still require further investigation, including (a) the therapeutic difference between the use of autologous *versus* allogeneic MT sources and the effect of the acquired heteroplasmy; (b) the effect of tissue sources and demographic donor variability; (c) the fate and persistence of the donated MT in the recipient cells; and (d) the identification of protein and RNA cargo shuttled by the transferred MT.

## Conclusions

MSCs, in their natural microenvironment, appear to be primarily glycolytic, but when entering a proliferative state they undergo a metabolic shift toward OXPHOS and increased mitochondrial activity. This OXPHOS metabolic state is associated with increased production of ROS and leads to mitochondria dysfunction. In contrast, MSC adipogenesis or osteoblast differentiation is associated with a decrease of the pentose phosphate pathway (PPP) pathway. In parallel, the mitochondrial enzymatic activities are increased with a high oxidative phosphorylation and beta-oxidation.

All the findings detailed in this review are compatible with the notion that MSCs respond to damaged or inflamed tissue through the transfer of MT to injured and immune cells, conveying a type of signaling that contributes to the restoration of cell homeostasis and immune function. The eventual reversal, induced by MT transfer, of the metabolic and pro-inflammatory profile, will open new avenues to the understanding of inflammatory diseases, their relation to both systemic and local risk factors, and also leads to new therapeutic tools for the control of the disease.

## Author Contributions

CJ and MK contributed equally to the review. All authors contributed to the article and approved the submitted version.

## Funding

This work was supported by grants from National Agency for Investigation and Development: ANID (Agencia Nacional de Investigación y Desarrollo) [FONDECYT regular #1170852, #1201420 and #1211749].

## Conflict of Interest

MK is the chief scientific officer of Cells for Cells and Regenero, the chilean consortium for regenerative medicine.

The remaining authors declare that the research was conducted in the absence of any commercial or financial relationships that could be construed as a potential conflict of interest.
